# Antimelanogenesis Effect of Methyl Gallate through the Regulation of PI3K/Akt and MEK/ERK in B16F10 Melanoma Cells

**DOI:** 10.1155/2022/5092655

**Published:** 2022-12-07

**Authors:** Zhi Jiao Cheng, Guo Fong Dai, Jue Liang Hsu, Jen Jie Lin, Wen Tung Wu, Ching Chyuan Su, Yu Jen Wu

**Affiliations:** ^1^Department of Beauty Science, Meiho University, Pingtung 91202, Taiwan; ^2^Yu Jun Biotechnology Co., Ltd., Kaoshiung, Taiwan; ^3^Department of Biological Science and Technology, National Pingtung University of Science and Technology, Pingtung, Taiwan; ^4^Research Center for Austronesian Medicine and Agriculture, National Pingtung University of Science and Technology, Pingtung, Taiwan; ^5^Department of Food and Nutrition, Meiho University, Pingtung 91202, Taiwan; ^6^Antai Medical Care Corporation Antai Tian-Sheng Memorial Hospital, Pingtung 928, Taiwan

## Abstract

Methyl gallate is a polyphenolic compound found in many plants, and its antioxidant, antitumor, antibacterial, and anti-inflammatory effects have been extensively studied. More recently, antidepressant-like effects of methyl gallate have been demonstrated in some studies. In the present study, we examined the effects of methyl gallate on melanogenesis, including the tyrosinase inhibitory effect, the melanin content, and the molecular signaling pathways involved in this inhibition. The results showed that methyl gallate inhibited tyrosinase activity and significantly downregulated the expressions of melanin synthesis-associated proteins, including microphthalmia-associated transcription factor (MITF), tyrosinase, dopachrome tautomerase (Dct), and tyrosinase-related protein-1 (TRP1). In conclusion, our findings indicated that activation of MEK/ERK and PI3K/Akt promoted by methyl gallate caused downregulation of MITF and triggered its downstream signaling pathway, thereby inhibiting the production of melanin. In summary, methyl gallate showed significant inhibitory activity against melanin formation, implying that it may be a potential ingredient for application in skin-whitening cosmetics.

## 1. Introduction

Melanocytes are responsible for the production of melanin, which contributes to the colored pigmentation in the skin and hair. In the epidermis of the skin, ultraviolet irradiation promotes melanocytes to generate melanin, and the dispersal of melanin via melanosomes to keratinocytes protects the human skin from extensive sunburn. Nonetheless, aberrant regulation of melanogenesis leads to pigmentation disorders such as melasma, hyperpigmentation, age spots, and blemishes [1–3]. Several plant-derived agents, such as kojic acid or arbutin (tyrosinase inhibitors), have been used for skin whitening, as they may regulate melanogenesis and are applicable in the treatment of hyperpigmentation [4].

Melanogenesis is a tyrosinase-initiated pigmentation process that converts L-3,4-dihydroxyphenylalanine to dopaquinone, followed by oxidation to form melanin [5]. The pathway of melanogenesis is largely controlled by the level and activity of tyrosinase [6]. Hence, inhibition of tyrosinase has been utilized for cosmetic purposes and skin bleaching. A number of signaling cascades, such as cAMP-related pathways, have been reported to have crucial roles in controlling melanogenesis [7, 8]. Adenylyl cyclase activation induced by UV upregulates the formation of cAMP, which sequentially binds to melanocortin receptor 1 (MC1R) in melanocytes, thus triggering cAMP production and protein kinase A (PKA) activation [9]. The process consequently leads to phosphorylation of the cAMP responsive-element binding protein (CREB), which in turn increases the transcription level of the microphthalmia-associated transcription factor (MITF) gene [10, 11]. MITF is an important transcription factor for key proteins involved in melanin synthesis in melanocytes that regulates the expressions of tyrosinase, tyrosinase-related protein-1 (TRP-1), dopachrome tautomerase (Dct), and PKC-*β* [12, 13].

MITF is a key factor in determining PKC-*β*- and cAMP-dependent signaling in melanogenesis regulation [14, 15]. Moreover, MITF is also a major element controlling DNA replication and the genome stability of melanoma cells via the downregulation of DNA alteration and inhibition of defects during cell proliferation [16, 17], while DOPA-chrome tautomerase reduces oxidative stress-induced cell injury and may regulate the sensitivity of melanoma cells towards certain chemotherapy drugs [18, 19].

In melanocyte cells, the mitogen-activated protein kinase (MAPK) family, including p38MAPK and extracellular signal-regulated kinase (ERK), is mostly involved in regulating MITF expression [20, 21]. In addition, phosphatidylinositol-3-kinase PI3K/Akt signaling has been recently identified as regulating melanogenesis. The upregulation of PI3K/Akt activates melanin synthesis, which is mediated by MITF expression and subsequent tyrosinase, TPR-1 and TRP-2 expressions [22, 23]. Thus, based on the findings of previous studies, these signaling pathways have the potential to be developed as a new strategic target for controlling melanin synthesis.

Methyl gallate is a gallic acid derivative and has been shown in previous studies to possess several biological properties, such as antioxidant [24], anti-inflammatory [25], antibacterial [26], and antitumor effects [27, 28]. Few studies have focused on the effects and molecular mechanism of methyl gallate against melanogenesis. Therefore, using an *in vitro* cell-culture model, this study aims to identify the antimelanogenesis mechanism of methyl gallate in B16F10 melanocyte cells.

## 2. Materials and Methods

### 2.1. Chemicals and Reagents

Methyl gallate, kojic acid, arbutin, protease inhibitor cocktail, MTT, 3,4-dihydroxyphenylalanine (L-DOPA), LY294002, PD98059, and goat antirabbit *β*-actin antibody were purchased from Sigma-Aldrich (St. Louis, MO, USA). PVDF (polyvinylidene difluoride) membranes were obtained from Millipore (Bellerica, MA, USA). Antibodies against ERK, *p*-ERK, TRP1, and Dct were obtained from ProteinTech group (Chicago, IL, USA). Antibodies against tyrosinase, MITF, *p*-MITF, MC1R, RSK1, *p*-RSK1 CREB, and *p*-CREB were purchased from Epitomics (Burlingame, CA, USA). Antibodies against PI3K, *p*-PI3K, AKT, *p*-AKT, p38MAPK, and *p*-p38MAPK were purchased from Cell Signaling Technology (Danvers, MA, USA).

### 2.2. Cell Culture and Treatment with Methyl Gallate

B16F10 mouse melanoma cells were purchased from the Taiwan Food Industry Research and Development Institute (Hsinchu, Taiwan). The cells were cultured and maintained in Dulbecco's modified Eagle's medium (DMEM; Gibco Life Technologies, Carlsbad, CA, USA) contained with 10% FBS, 100 U/mL penicillin, and 100 *μ*g/mL streptomycin at 37°C with 5% CO_2_ atmosphere. Methyl gallate dissolved in DMSO and further diluted with DMEM to achieve the indicated final concentrations (50, 100, 200, 300, 400, 500, and 600 *μ*M). Cells were cultured with different concentrations of methyl gallate and harvested after 24 h of incubation. Experiments were performed in triplicate and repeated multiple times.

### 2.3. Cell Viability Assay

MTT cell viability assay was assessed to determine the viability of methyl gallate against B16F10 cells. Cells were seeded at 1 × 10^4^ cells/well in 96 well plates. After treatment with various concentrations of methyl gallate, the cells were incubated at 37°C for 24 h. The cell viability was determined using 3-(4,5-cimethylthiazol-2-yl)-2,5-diphenyl tetrazolium bromide (MTT) solution (1 mg/ml, 50 *μ*l/well) and then added to each well, and cells were incubated at 37°C for 4 h. After the removal of the MTT solution, 100 *μ*l DMSO was added to the well and incubated for 10 min. Absorbance was determined at 595 nm using a microtiter plate ELISA reader.

### 2.4. Melanin Content Analysis

B16F10 cells (1 × 10^5^ cells/well) were incubated in 24 well plates. In brief, B16F10 cells were treated with methyl gallate for 24 h. The cells were washed with PBS and then dissolved in 200 *μ*l of 1N NaOH at 80°C for 2 h. The samples were centrifuged for 30 min at 12,000 rpm to collect the supernatant. The relative melanin content was determined by measuring the absorbance at 475 nm on a microtiter plate ELISA reader. The melanin content was determined using standard curves using synthetic melanin solutions (0∼200 *μ*g/ml). Melanin production was calculated as the *μ*g of melanin/*μ*g of total proteins in a cell extract.

### 2.5. Tyrosinase Activity Assay

Tyrosinase activity was estimated as L-DOPA oxidase activity. B16F10 cells (2 × 10^6^ cells/well) were incubated in 24 well plates. After treatment with different concentrations of methyl gallate (0, 10, 50, 100, and 200 *μ*M) for 24 h, cells were washed with PBS and lysed with RIPA buffer containing protease inhibitor cocktail. The cell lysates were then centrifuged at 12,000 rpm for 15 min to collect the supernatant. The sodium phosphate buffer (0.1 M, pH 7.0) reacted with an equal volume of 1 mg/ml L-DOPA. After incubation at 37°C for 2 h. Absorbance was then measured at 405 nm in an ELISA reader. Each measured result was expressed as the percentage change from the control [12].

### 2.6. Tyrosinase Activity Staining

The tyrosinase activity staining was analyzed according to a previously described method [12]. B16F10 cells were incubated in 10 cm dish plates. In brief, the cells were lysed with RIPA buffer containing protease inhibitor cocktail. The total protein concentration was determined with Bradford Assay (Bio-Rad, Hercules, CA, USA), after which 25 *μ*g of total protein were loaded onto and separated by 10% SDS-PAGE. The sample was prepared in 0.1% SDS and *β*-mercaptoethanol and heat treatment was avoided. After electrophoresis, the gels were soaked in 10 mM Na_2_HPO_4_ buffer (pH 6.2) for 30 min, followed by incubation in the same buffer containing 2 mM L-DOPA at 37°C.

### 2.7. Protein Extraction and Western Blotting

After treatment, B16F10 cells were lysed with RIPA buffer containing protease inhibitor cocktail. Equal protein amounts (25 *μ*g) extracted from whole cells were loaded onto and separated by 12.5% SDS–PAGE and transferred to a polyvinylidene difluoride (PVDF) membrane (Millipore). The membranes were blocked with PBST buffer (PBS buffer containing 0.05% Tween 20) containing 0.2% gelatin for 2 h at 4°C. Thereafter, the membranes were incubated with appropriate rabbit polyclonal antibodies against mouse tyrosinase, TRP-1, Dct, MC1R, MITF, *p*-MITF, CREB, *p*-CREB, p38MAPK, *p*-p38MAPK, ERK, *p*-ERK, MEK, *p*-MEK, PI3K, *p*-PI3K, AKT, *p*-AKT, RSK1, and *p*-RSK1 for overnight at 4°C. The membranes were washed three times in PBST and then probed with goat antirabbit horseradish peroxidase-conjugated antibody (1 : 5,000) for 1 h. All bands were visualized using ECL western blotting reagents (pierce). The western blot data were quantified with Image J 1.47 software (https://www.downloadcrew.com/article/28008-imagej).

### 2.8. Statistical Analysis

The results of the MTT assay were subjected by Student's *t*-test (Sigma-Stat 2.0, San Rafael, CA, USA). Results with *p* < 0.05 were considered statistically significant.

## 3. Results

### 3.1. Effect of Methyl Gallate on B16F10 Melanoma Cells by Cell Viability Assay

**Figure 1:**
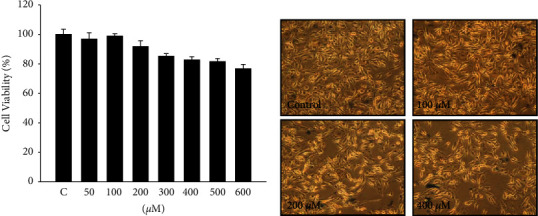
Cell viability of methyl gallate-treated B16F10 cells. (a) B16F10 cells were treated with methyl gallate of a series of concentrations, and the cell viability was determined by MTT assay. (b) The morphological change of B16F10 cells after methyl gallate treatment. (100× magnification).

Cells were cultured in methyl gallate of varying concentrations, ranging from 50 to600 *μ*M, and the cell viability was measured by MTT assay. We also used kojic acid (200 *μ*M) and arbutin (2 mM) as positive controls to compare the survival rates of B16F10 cells. The experimental results showed that the survival rate of the B16F10 cells was approximately 90% when 200 *μ*M methyl gallate was added and approximately 80% when 600 *μ*M methyl gallate was added and with 200 *μ*M kojic acid and 2 mM arbutin separately. The cell viability was approximately 80% in each experiment (Figure 1(a)). We also observed the cell type using a microscope and found that when up to 400 *μ*M methyl gallate was added, there was no significant change in cell type (Figure 1(b)).

### 3.2. Tyrosinase Activity and Melanin Production in B16F10 Melanocytes after Methyl Gallate Treatment

**Figure 2:**
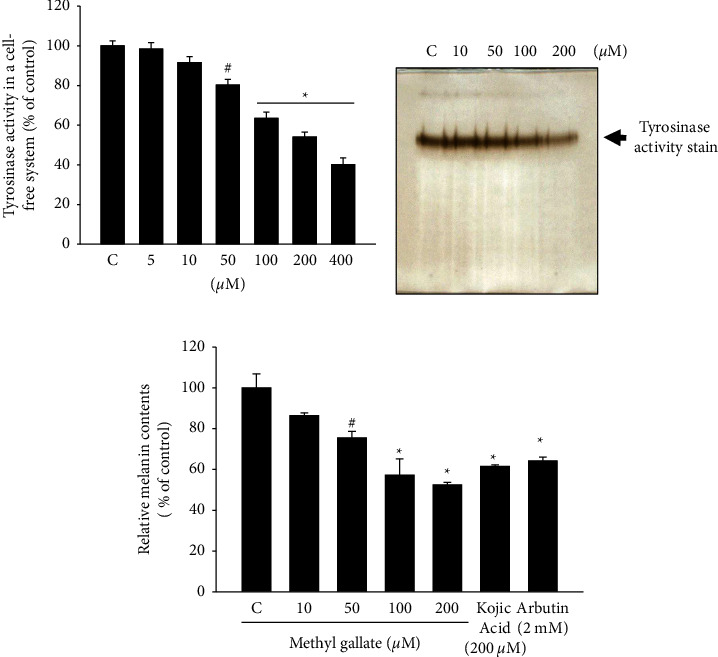
Effects of methyl gallate on tyrosinase activity and melanin production in B16F10 cells. Cells were incubated with 5, 10, 50, 100, 200, and 400 *μ*M methyl gallate for 24 h, and the tyrosinase activity and melanin content in the cells were measured. (a) Tyrosinase enzyme activity assay, (b) activity of tyrosinase detected in polyacrylamide electrophoresis gel after incubation with 3,4-dihydroxyphenylalanine, (c) melanin levels in cells after methyl gallate treatment. Data presented as percentage of the control. ^#^*p* < 0.05, ^*∗*^*p* < 0.01.

We used an *in vitro* tyrosinase activity assay to study the inhibitory effect of methyl gallate. The results showed that 100 *μ*M or higher concentrations of methyl gallate significantly reduced the tyrosinase activity as compared with the controls; treatment with 200 *μ*M methyl gallate was observed to inhibit tyrosinase activity by about 50% (Figure 2(a)). In addition, we used tyrosinase activity gel stain analysis to confirm the finding. Following the addition of different concentrations of methyl gallate (10, 50, 100, and 200 *μ*M), tyrosinase activity staining analysis showed that with the increase of methyl gallate concentration, the inhibition of tyrosine activity was greater (Figure 2(b)).

Subsequently, we examined melanin synthesis in B16F10 melanocytes. When the cells were treated with 50 *μ*M or higher concentrations of methyl gallate, there were significant differences in melanin synthesis as compared with the control. When 200 *μ*M methyl gallate was added, melanin synthesis was inhibited by approximately 50%. In addition, we used positive control groups treated with 200 *μ*M kojic acid and 2 mM arbutin for inhibition of melanin synthesis and observed that 100 *μ*M methyl gallate had a better effect than kojic acid and arbutin, while 200 *μ*M methyl gallate was even more effective (Figure 2(c)).

### 3.3. Effect of Methyl Gallate on Melanin Synthesis-Related Pathway Proteins

**Figure 3:**
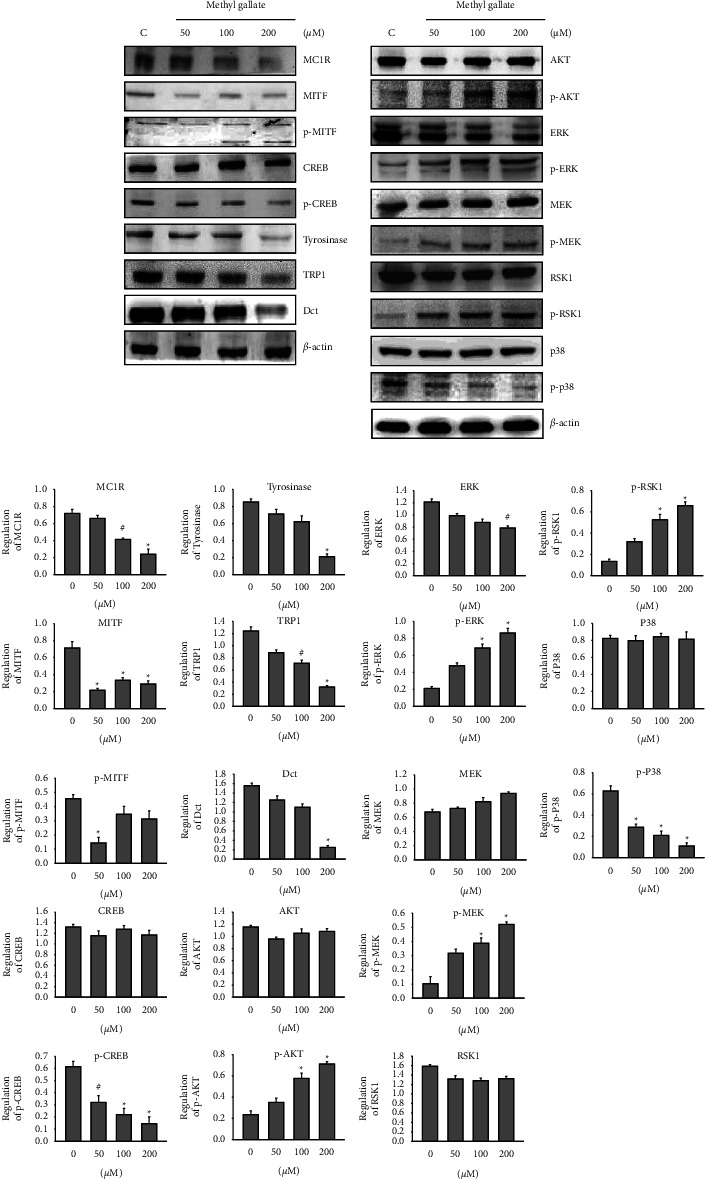
Western blotting analysis of melanogenesis-associated proteins in B16F10 cells treated with methyl gallate. (a) Antibodies against a series of melanogenesis-associated proteins as indicated were utilized to detect the protein expression levels in cells treated with 50, 100, and 200 *μ*M methyl gallate. An antibody against *β*-actin was used as the protein loading control. (b) Quantification of western blot bar graphs with *β*-actin (C control). ^#^*p* < 0.05, ^*∗*^*p* < 0.01.

From the above experimental results, it was found that methyl gallate inhibited the activity of intracellular tyrosinase and melanin production. To understand whether it also affects the expressions of other melanin synthesis-related proteins, we used different concentrations of methyl gallate (50, 100, and 200 *μ*M) for treatment. After 24 hours of reaction, western blotting analysis was used to determine differences in protein expressions. From the experimental results, it was observed that the expressions of melanin-related proteins, such as MC1R, were decreased with increasing methyl gallate concentrations; decreases in the expressions of MITF, phosphorylated CREB, tyrosinase, TRP-1, and Dct were also observed with increasing methyl gallate concentrations (Figure 3).

p38MAPK, MEK/ERK, and PI3K/Akt are known to be associated with melanin synthesis-related pathways. The expression levels of these proteins after the addition of methyl gallate are shown in Figure 3. The expressions of *p*-MEK, *p*-ERK, *p*-RSK1, and *p*-AKT increased with an increasing concentration of methyl gallate, while *p*-p38MAPK was downregulated, and the expressions of MEK, ERK, RSK1, and Akt did not change (Figure 3). Therefore, it was speculated that methyl gallate may also affect B16F10 melanocytes melanogenesis through the activation of MEK/ERK and PI3K/Akt signaling pathways.

### 3.4. Effects of MEK/ERK and PI3K/Akt Inhibition on Methyl Gallate-Repressed Melanogenesis-Related Protein Expressions

**Figure 4:**
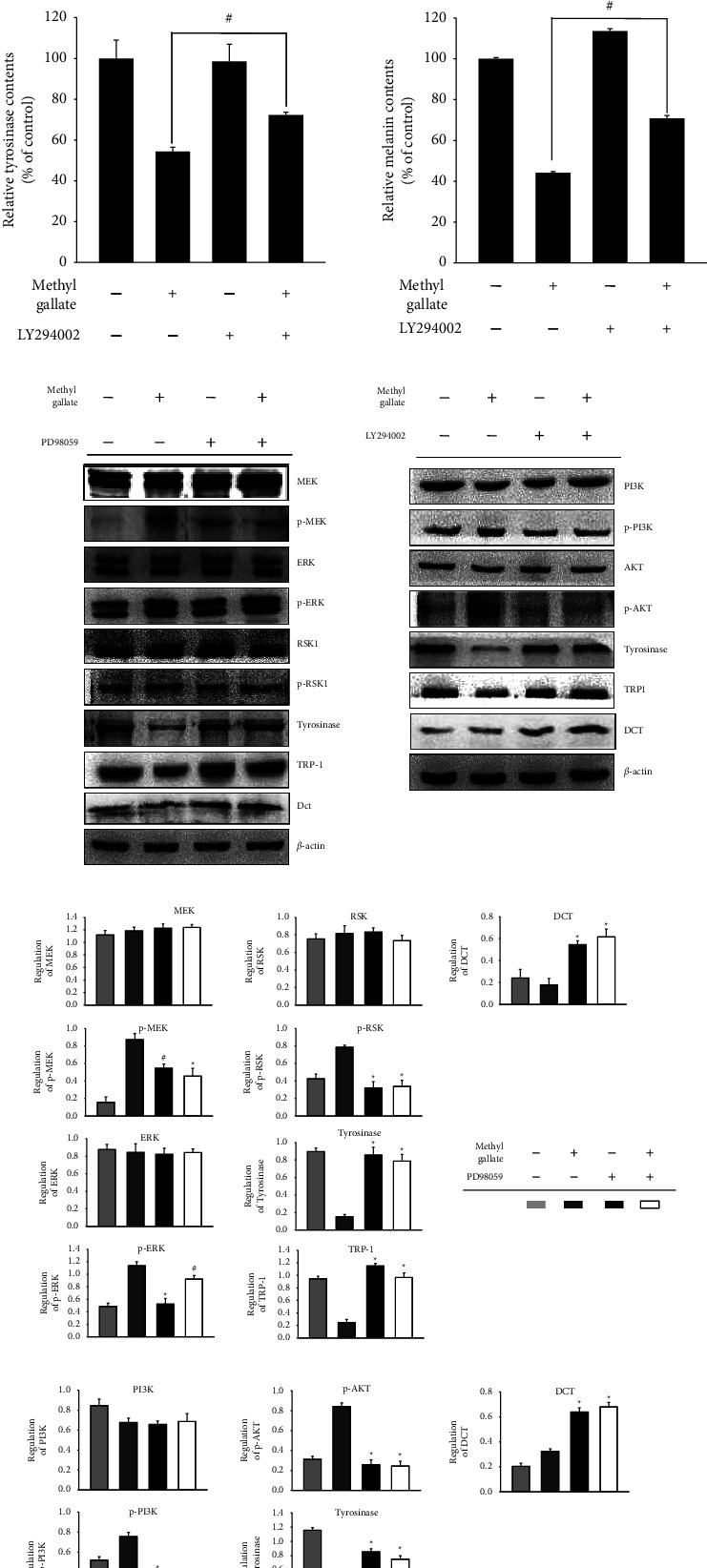
The recovery effect of inhibition of PI3K/Akt or MEK/ERK on tyrosinase activity, melanin content, and melanogenesis-associated protein expressions in B16F10 cells treated with methyl gallate. B16F10 cells were pretreated with/without 20 *μ*M PD98059 or LY294002 for 1 hr, followed by incubation with 200 *μ*M methyl gallate for 24 hr. (a) Melanin content and tyrosinase activity in the cells. Data are presented as percentage of the control, and the results were obtained from a representative of three independent experiments. An antibody against *β*-actin was used as the protein loading control. (b) Western blotting analysis of melanogenesis-associated protein expressions. (c) Quantification of western blot bar graphs with *β*-actin (C control). ^#^*p* < 0.05, ^*∗*^*p* < 0.01.

After adding MEK/ERK and PI3K/Akt inhibitors (PD98059 and LY294002, respectively), the activity of tyrosinase and the content of melanin in the cells were studied. It was found that the activity of tyrosinase and the melanin content decreased significantly by approximately 50% after methyl gallate treatment in B16F10 melanocytes (Figure 4(a)). To confirm whether methyl gallate inhibited melanin synthesis through PI3K/Akt and MEK/ERK, we employed western blotting to examine melanin synthesis-related protein expressions in MEK/ERK and PI3K/Akt signaling. The results showed that the inhibition of MEK/ERK and PI3K/Akt led to higher expressions of tyrosinase, TRP-1, and Dct than methyl gallate treatment alone. Both PD98059 and LY294002 treatments had significant effects in terms of the degree of recovery of MITF, tyrosinase, TRP-1, and Dct protein expressions as compared with cells treated with methyl gallate only. These results demonstrated that methyl gallate activates the MEK/ERK and PI3K/Akt signaling pathways, which in turn affect melanin synthesis in B16F10 cells (Figure 4(b)).

## 4. Discussion

Methyl gallate is a polyphenolic compound derived from plants and has been reported to possess a variety of bioactivities. In this study, we investigated the molecular mechanism of methyl gallate inhibition of melanin production in melanocytes. According to the results of cell-survival experiments, 0–600 *μ*M methyl gallate had no significant cytotoxicity against B16F10 melanocytes. In addition, methyl gallate treatment caused significant dose-dependent inhibition of intracellular tyrosinase and melanin contents (Figure 1).

Methyl gallate at 200 *μ*M significantly inhibited the activity of tyrosinase *in vitro* and also inhibited the tyrosinase activity in a staining experiment. Kojic acid and arbutin (a tyrosinase inhibitor) are used in the treatment or prevention of abnormal skin pigmentation [29, 30], The experimental results showed that the cell viability following the treatment with 200 *μ*M methyl gallate was higher than that with the treatment with 200 *μ*M kojic acid and 2 mM arbutin, and the inhibition of melanin by 200 *μ*M methyl gallate was better than that of 200 *μ*M kojic acid and 2 mM arbutin. Methyl gallate is a potent compound that inhibits melanin and was proven to be more effective than kojic acid and arbutin in cell experiments.

In addition to the cAMP regulatory pathway as the major signal transduction pathway, activation of MEK/ERK and PI3K/Akt signaling pathways (also) regulates melanin synthesis [12, 31]. The phosphorylated p38MAPK activates MITF to ultimately stimulate melanin synthesis, while the activation of ERK 1/2 and JNK leads to a decrease in melanogenesis via MITF degradation [32].

Study results have indicated that the MEK/ERK pathway regulates the phosphorylation of MITF. When the MEK/ERK pathway is activated, it will promote the phosphorylation of MITF, followed by ubiquitination, and degradation. The downstream expressions of melanin-related proteins such as tyrosinase, TRP1, and Dct are decreased, reducing melanin synthesis [33, 34]. Activation of the PI3K/Akt signaling pathway is therefore subjected to a strictly-regulated signal-dependent approach, which in turn affects the phosphorylation of MITF and regulates melanin biosynthesis [22, 35]. In addition to transcriptional regulation, MITF is also phosphorylated by various post-translational modifications, in addition to ERK; in particular, ribosomal S6 kinase (RSK) and glycogen synthase kinase-3*β* (GSK3*β*) [11] have been used to phosphorylate MITF.

The phosphorylated active CREB further binds MITF, which in turn stimulates the transcription of the key melanogenic enzymes [36]. CREB phosphorylation induces transcription of MITF. The *p*-CREB expression decreased after treatment with methyl gallate, and *p*-MITF protein fragmentation increased with increasing concentrations of methyl gallate, as did the expressions of *p*-MEK and *p*-ERK. The protein expression levels of *p*-RSK1 and *p*-AKT increased significantly. It was speculated that methyl gallate may activate *p*-MITF via the MEK/ERK and PI3K/Akt signaling pathways to down-regulate tyrosinase, TRP-1, and DCT in B16F10 melanocytes [12].

Previous studies have demonstrated that melanogenesis is mediated by the regulation of MITF activation via phosphorylation of p38 MAPK [10]. Activation of the p38 MAPK signaling pathway increases the transcription of tyrosinase, which activates melanin synthesis [37]. In this study, methyl gallate inhibited the phosphorylation level of p38MAPK, which could participate in and inhibit melanin production.

We used a MEK/ERK inhibitor (PD98059) and a PI3K/Akt inhibitor (LY294002) to verify whether methyl gallate activates the MEK/ERK and PI3K/Akt signaling pathways, which in turn affects tyrosinase activity and melanin synthesis and decreases the expressions of proteins involved in melanin synthesis. First, we added a MEK/ERK inhibitor (PD98059) and a PI3K/Akt inhibitor (LY294002) and recorded the activity of tyrosinase and the content of melanin. Then, we used immunostaining analysis to verify the melanin synthesis pathways of MEK/ERK and PI3K/Akt. The results suggested that methyl gallate does activate the MEK/ERK and PI3K/Akt signaling pathways, thereby reducing the expressions of tyrosinase and melanin-related proteins and inhibiting the synthesis of melanin [12].

Nerya et al. isolated [6]-Shogaol from ginger and showed inhibited melanogenesis through the activation of extracellular responsive kinase (ERK) and phosphatidylinositol-3-kinase- (PI3K/Akt-) mediated MITF degradation [38]. Ellagic acid inhibits melanin, tyrosinase, and TRP-1/-2 by downregulating CREB/MITF expression through JNK, ERK, and AKT signaling pathways in *α*-MSH-stimulated B16F10 cells [39].

The previous study showed that methyl gallate decreased melanin pigmentation in a concentration-dependent manner but did not directly inhibit tyrosinase activity. Further analysis showed that methyl gallate had no effect on extracellular signal-regulated kinase (ERK) activation but induced phosphorylation of glycogen synthase kinase 3*β* (GSK3*β*) [40]. The results of the current study differed from the previous findings, in that our results validated the ability of methyl gallate to inhibit melanin synthesis.

## 5. Conclusion

We demonstrate that methyl gallate suppresses the tyrosinase activity in B16F10 cells and reveal the molecular mechanism involved in melanogenesis. Our data suggest that methyl gallate may regulate melanogenesis by restraining the production of MITF, tyrosinase, TRP1, and Dct, and the process is associated with the phosphorylation of PI3K/Akt or MEK/ERK. According to our results, methyl gallate reduces melanin synthesis and could be a useful agent for skin whitening; it may also have the potential for application in cosmeceuticals in the future.

## Data Availability

Data generated or analyzed during this study are provided in full within the published article.
